# Letter to the Editor re: “Systematic review and meta-analysis comparing Manta device and Perclose device for closure of large bore arterial access.” *J Vasc Access*. 2024 Jan 8:11297298231222314

**DOI:** 10.1177/11297298241254255

**Published:** 2024-06-01

**Authors:** Angela Di Giorgio, Claudia Carnuccio, Antonio Nesci, Alessia D’Alessandro, Angelo Santoliquido

**Affiliations:** 1 Department of Cardiovascular Sciences, Angiology and Non-invasive Vascular Diagnostics Unit, Fondazione Policlinico Universitario Agostino Gemelli IRCCS, Rome, Lazio, Italy; 2 Catholic University of the Sacred Heart, Rome, Italy

Dear Editor,

We read with great enthusiasm the article of Tayyab Cheema et al. that compared Manta device and Perclose device for closure of large bore arterial access.^1^

Vascular closure devices (VCDs) have gained consideration due to the increasing use of large bore arterial accesses and have been shown to be non-inferior to manual compression in achieving successful hemostasis. Actually, there is no consensus on the recommended device of choice^2^ and, to date, there were no studies comparing their effectiveness, except for the distinguished work of Tayyab Cheema et al. Authors reviewed and performed a meta-analysis focusing on the safety and effectiveness profiles of MANTA and Perclose devices, not revealing significant differences.

Briefly, Perclose ProGlide (Abbott Vascular, Santa Clara, CA) achieves hemostasis with a single 3-0 polypropylene monofilament suture at the vascular access site with a 92% success rate and a 4.4% complication rate.^3^ MANTA (Teleflex, Wayne, PA) consists of an extravascular hemostatic collagen structure held in place by an endoluminal molded polymer toggle, stainless steel element, and suture that achieves high technical efficacy with a low risk of vascular complications.^4^

We agree in saying that, bleeding, deployment failure, and device migration are the most common complications associated with VCDs. Color-Doppler ultrasound guides intraoperative VCD deployment and allows early detection of intra- and postoperative adverse events; its use is advocated at each step of the procedure to promptly identify VCD-related complications. In this regard, we need to emphasize that vascular ultrasound is a valuable tool with the advantages of being fast, non-invasive, and readily available, although it requires an experienced operator. In fact, recent meta-analytic data derived from four randomized clinical trials brightly reported that ultrasound guidance for transfemoral access might be an effective and simple choice in order to reduce major vascular complication and major bleeding.^5^ Vascular ultrasound identifies VCDs immediately after placement and helps in diagnosing complications in the peri-operative follow-up.

**Figure 1.**
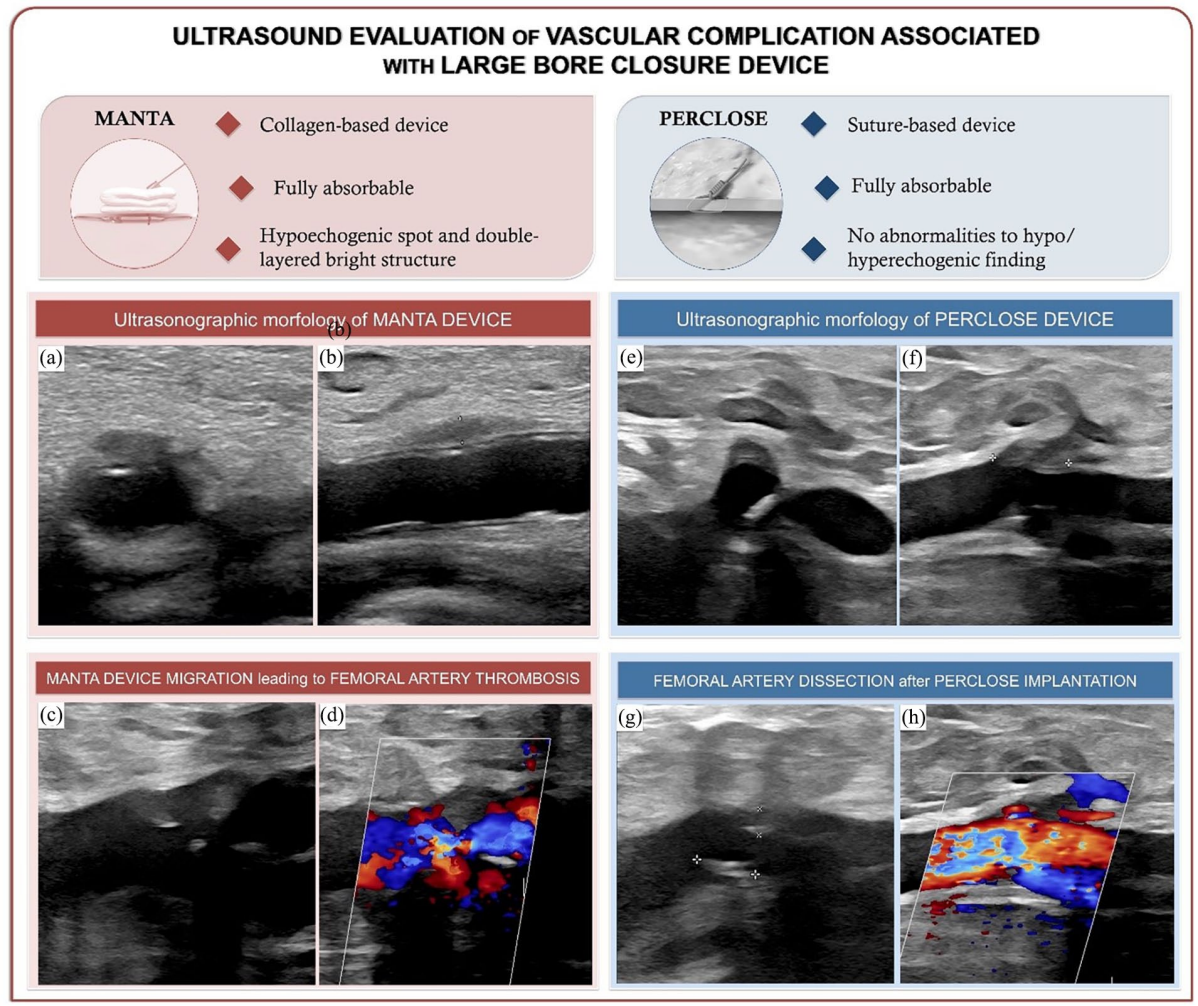
Ultrasound evaluation of large bore closure devices’ vascular complications. Common femoral artery (CFA)’s transverse (a) and longitudinal (b) ultrasound (B-mode) images showing MANTA’s normal ultrasound characteristics (a double-layered bright structure in the lumen formed by the poly-lactate toggle, and a perivascular hypoechogenic area corresponding to the collagen placement). Longitudinal ultrasound image (B-mode) (c) showing MANTA displacement leading to partial obstructive CFA thrombosis and hemodynamic stenosis on color Doppler ultrasound (d). Transverse (e) and longitudinal (f) ultrasound (B-mode) images showing no significant abnormalities of the CFA vessel wall in correspondence with Perclose, except for a hypoechoic shadow in the surrounding tissue, compatible with the introducer track. In presence of vessel diffuse atherosclerosis and vascular calcifications, the use of Perclose has resulted in a CFA dissection, with a visible intimal flap (highlighted in the image by cursors) (g) and acceleration of blood flow at this level on the color Doppler ultrasound image (h).

Moreover, the normal ultrasonographic appearance of VCDs could resemble the presentation of their complications, hence the need for an evaluation by an experienced operator and our willingness to focus on their uncomplicated appearance through our images (Figure 1). We suggest integrating ECD assessment into procedural planning and performing short-term post-procedural evaluations to promptly identify VCD-related complications. Long-term vascular ultrasound studies are essential to assess the impact of these devices on the arterial wall over time, including potential remodeling and stenosis. It is also critical to evaluate the role of VCDs as potential points of least resistance for future endovascular procedures on the same artery.
